# Identification of Au-hydrides as key intermediates in the reduction of Au(iii) prodrugs to active Au(i) species under protic conditions

**DOI:** 10.1039/d5sc06212h

**Published:** 2026-01-07

**Authors:** Jasmine Ochs, Nils Metzler-Nolte

**Affiliations:** a Faculty of Chemistry and Biochemistry, Inorganic Chemistry I – Bioinorganic Chemistry, Ruhr University Bochum Universitaetsstrasse 150 Bochum 44801 Germany Nils.Metzler-Nolte@rub.de

## Abstract

Similar to Pt^IV^ prodrugs, Au^III^ anticancer complexes are believed to undergo intracellular reduction, thereby gaining their activity from the resulting Au^I^ species. Unlike for Pt^IV^, the underlying mechanism of this process remains poorly understood for Au^III^. To elucidate this mechanism, we investigated the reaction of [Au(ppy)Cl_2_], a model Au^III^ complex (ppy: phenylpyridine), with two biologically relevant reductants: lipoic acid (lpa) and *N*-acetyl-l-cysteine-methyl ester (NAC-OMe). Our findings reveal that lpa transfers a hydride to the Au, while cysteine derivatives only bind to the metal. The Au–H complex, even visible in protic solvents by NMR spectroscopy, produced by lpa is essential for enabling a sequence of oxidative addition and reductive elimination reactions that lead to Au^I^ species eventually. These observations provide valuable insights into the mechanisms by which anticancer gold drug candidates are reduced within the cell.

## Abstract 2

Au^III^ anticancer complexes undergo intracellular reduction to the active Au^I^ species. Here, we identify Au hydrides as important intermediates, and demonstrate the importance of reductive elimination reactions as well as polymetallic Au clusters.

## Graphical abstract

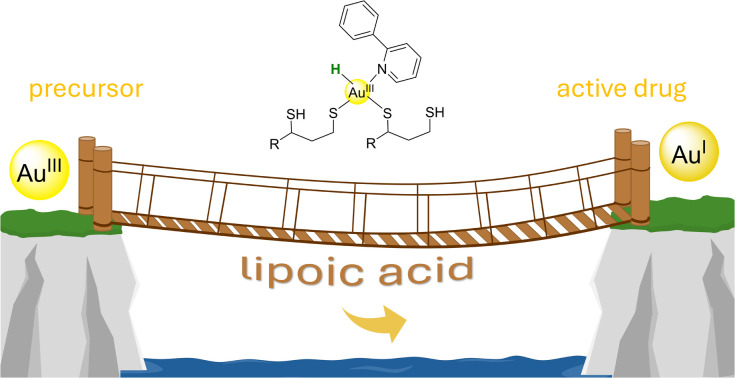

## Introduction

Since cisplatin initiated the success of Pt^II^ compounds in chemotherapy, research has focused on improving the drug to minimize toxic side effects.^1^ An example for such an improvement is the development of Pt^IV^ prodrugs with the metal in the higher +IV oxidation state. These are slowly reduced intracellularly to their active Pt^II^ counterparts, releasing two ligands in the process.^2^ Numerous studies have identified sodium ascorbate, l-methionine, l-cysteine and of course glutathione (GSH) as the primary intracellular reductants.^3^ Sodium ascorbate, a two-electron reductant, can reduce Pt^II^ either through an inner-sphere or outer-sphere mechanism.^4^ In the case of GSH, it is hypothesized that the reductive elimination of the axial ligands is facilitated by the formation of a bridge between thiol and coordinated acetate.^5^

Another approach to circumvent toxic side effects and resistances is the use of Au^I^ and Au^III^ compounds, which are isoelectronic and structurally similar to the Pt^II^/Pt^IV^ system.^6^ However, the high oxidation state must be stabilized for biological applications, usually using phosphine,^7^*N*-heterocyclic carbenes,^8^ or polydentate ligands featuring C and N binding sites.^9^ Gold complexes exhibit different modes of action, such as coordination to specific enzymes like thioredoxin reductase,^10^ and covalent modifications of the enzyme by Au^III^ catalyzed cross coupling reactions,^11^ making them a promising new class of compounds. Beside the binding to specific targets, it is assumed that intracellular reduction by ascorbate or thiols (as for Pt) activates the Au^III^ complexes to their biologically active Au^I^ analogues.^12^ For example, Che and co-workers employed an Au^III^ complex that releases its chelating ligand upon reduction, due to a change to linear geometry. Thereby, the fluorescent ligand is activated and the cytotoxic Au^I^-NHC complex bearing a GSH ligand is formed.^13^ Another possibility is the release of free Au^I^ ions from polypyridyl Au^III^ complexes, which leads to inhibition of thioredoxin reductase (TrxR), even under hypoxic conditions. Additionally, the ligand exhibits a second mode of action by binding to potassium channels, thereby damaging the lysosomal membrane.^14^ Interestingly, almost nothing is known about the actual reduction pathway, but cysteine and especially GSH are assumed to facilitate the reduction. Recent work by Mironov and Kharlamova^15^ on N^N bidentate Au complexes suggests ligand substitution, followed by “inner-sphere reduction” and the formation of polymeric Au^I^-thiolate species as a possible pathway. However, no intermediates could be isolated in this work, and the exact mechanism of reduction remains elusive. The analyzed N^N gold complexes are very prone to reduction, also rapidly leading to ligand loss. Notably, in these cases, the anticancer activity of such complexes is attributed to the ligand itself rather than the Au center.^12,16^ In contrast, highly stabilized Au^III^ complexes bearing bidentate C^N ligands bridged by CO, CH_2_, or NH groups also undergo first a ligand exchange but afterwards, in the presence of a sulphur or selenium containing amino acid, a C–S/Se cross coupling with the C^N ligand was observed, releasing free Au^I^.^17^ However, this process seems to be specific for the ligand type used in this work, and no intermediates were identified.

**Scheme 1:**
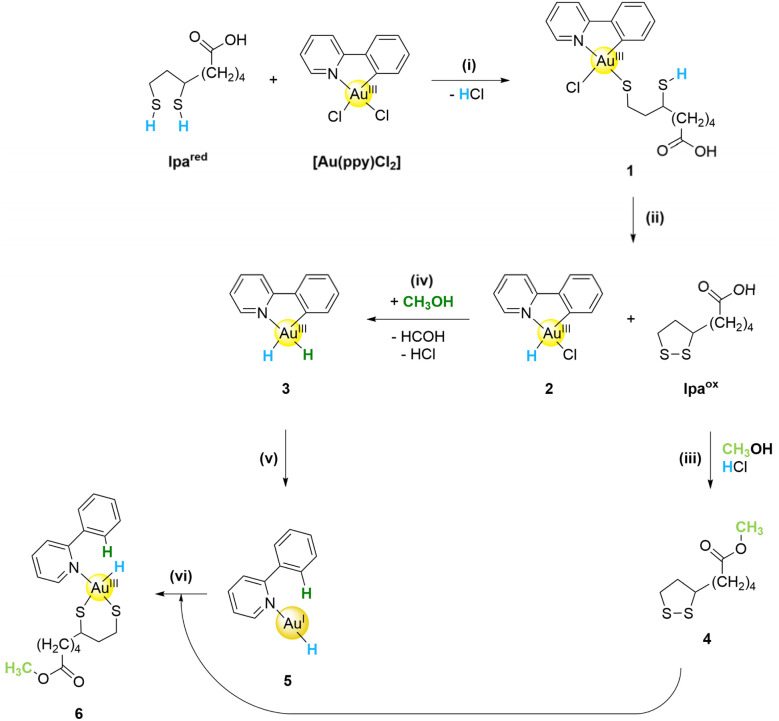
Proposed mechanism (part I) for the reaction between reduced lipoic and Au(ppy)Cl_2_ in DCM/MeOH. For clarity, all lpa protons are marked in blue and methanol is marked in green. (i) ligand exchange reaction with HCl formation, (ii) hydride transfer from the lipoic acid to the Au^III^-center under formation of the oxidized lipoic acid, (iii) esterification with methanol due to the acidic conditions, (iv) methanol oxidation with hydride transfer to Au^III^ generating a dihydride species, (v) reductive elimination of C–H with the H in the *cis* position to the carbanion, and (vi) oxidative addition of the oxidized lipoic acid.

**Fig. 1:**
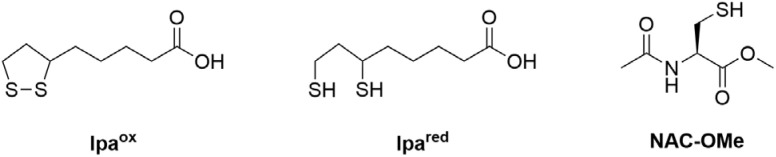
Structure of lipoic acid in the cyclic, oxidized form (left), the reduced species with two thiols (middle), and *N*-acetyl-l-cysteine methyl ester (NAC-OMe) (right).

To gain a more general overview of the mechanism of reduction, we present here a study on a stabilized Au^III^ model complex with two naturally relevant reductants. We selected Au^III^-dichlorido-phenylpyridine ([Au(ppy)Cl_2_]) (see Scheme 1 for its chemical formula) as a member of the cytotoxic bidentate C^N complexes, known for its stable +III oxidation state.^18^ For the biologically occurring reductants (see Fig. 1), we used *N*-acetyl-l-cysteine methyl ester (NAC-OMe) as a simplified analogue of GSH, and lipoic acid (lpa). Lipoic acid, like GSH, is an organosulfur compound, but it contains two thiol groups capable of forming a five-membered ring.^19^ Its unique structure facilitates substrate channeling between various active sites in multienzyme complexes, making it an essential cofactor for the citric acid cycle.^20^ Additionally, its low redox potential makes it a powerful reductant, capable of scavenging free reactive oxygen species (ROS). This dual functionality significantly contributes to maintaining a balanced redox system intracellularly.^21^ Using this model system, we were able to gain a deeper understanding of the reduction mechanism of Au^III^ complexes.

## Results and discussion

### Reaction with lipoic acid

First, we utilized naturally occurring lpa, known for its antioxidant properties. To address the poor water solubility of the Au precursor, reactions were performed in a DCM/MeOH (9 : 1) mixture. A further advantage of this solvent is the slower reaction rate compared to water, which facilitates the analysis of intermediates. Additionally, the solvent was degassed to prevent the oxidation of the dithiol to the corresponding disulfide.

The reaction between [Au(ppy)Cl_2_] and reduced lpa resulted in the formation of gold nanoparticles, visible as purple colloids in the mixture, as well as elemental gold as a side product, indicating the potential of lpa for Au^III^ reduction. Furthermore, various molecular Au compounds were detected as intermediates. After silica column purification of various batches, three compounds were isolated and analyzed by NMR spectroscopy and mass spectrometry.

**Fig. 2:**
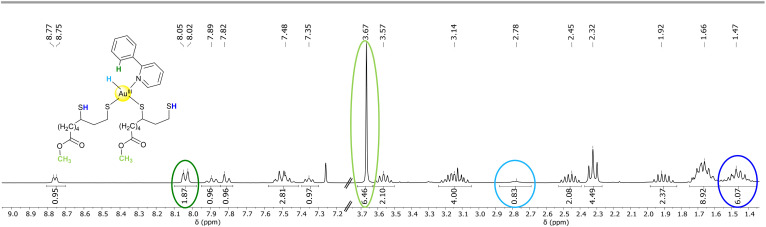
^1^H NMR of the reaction between lpa-H_2_ and [Au(ppy)Cl_2_] recorded in CDCl_3_. All unexpected signals are marked with circles and are assigned in the corresponding color inside the proposed structure.

**Fig. 3:**
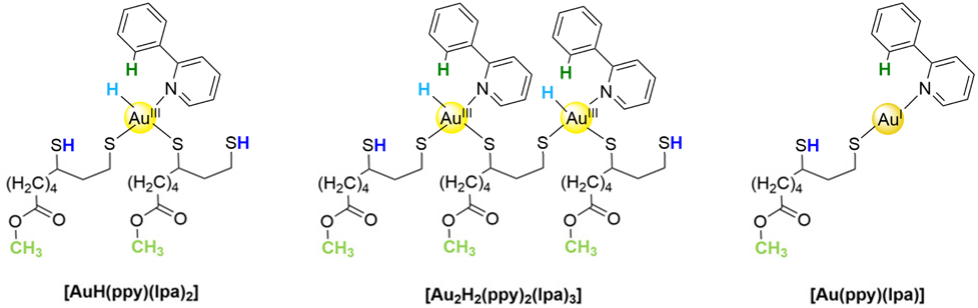
All observed gold complexes from the reaction of lpa^red^ and [Au(ppy)Cl_2_] with color code for NMR spectra analysis.

The most often observed complex is [AuH(ppy)(lpa)_2_], which has a 1 : 2 stoichiometry of ppy to lpa, as evident in the ^1^H NMR spectrum (see Fig. 2). The ^1^H NMR spectrum reveals the presence of two non-coordinating thiols, appearing as a multiplet at 1.47 ppm, which were further confirmed using Ellmann's reagent. Additionally, the singlet at 3.67 ppm indicates the esterification of lpa by methanol. The required HCl is released during lpa binding to gold, and because the reaction is carried out in a DCM/methanol mixture, it is non stabilized and, consequently, highly reactive. However, this reaction is not physiologically relevant, but rather an artifact caused by the presence of methanol in the solvent mixture, which is necessary for precursor solubility. An (unexpected) additional aromatic proton signal is observed at 8.03 ppm, showing the breakage and protonation of the Au–C bond, while ppy is still coordinated through the nitrogen atom. The fourth binding site is not any more occupied by a chlorido ligand, as evidenced by a negative “Purple of Cassius” test. This test is specific for Au^III^-chlorides, which are reduced by Sn^II^ chloride to form a purple pigment. Instead, the last ligand is an Au hydride, visible as a broad signal at 2.78 ppm. This hypothesis is supported by data from the Bochmann group which have isolated and confirmed the existence of Au^III^-hydrides with bi- and tridentate C- and N-cyclometallating ligands.^22,23^ They demonstrated that such hydrides occur in the ^1^H NMR spectra over a wide range from −8.5 ppm up to 7.0 ppm, which aligns well with our findings. All other data (including 2D COSY-NMR, see SI) confirm the proposed structure of [AuH(ppy)(lpa)_2_] as shown in Fig. 3.

The second isolated complex seems to be an intermediate structure, which is quite similar to [AuH(ppy)(lpa)_2_]. The striking difference between both complexes is the ratio of ppy and lpa as detected in the ^1^H NMR spectrum (see SI). The ratio of two ppy to three lpa ligands clearly shows the formation of the bridged binuclear complex [Au_2_H_2_(ppy)_2_(lpa)_3_], as depicted in Fig. 3 (middle). Apart from the altered stoichiometry, the NMR spectrum exhibits the same features, including the Au-H signal. Additionally, the structure was further confirmed by a signal in the ESI-MS at *m*/*z* = 685.2, which corresponds to the doubly protonated species.

Interestingly, a third intermediate was identified experimentally as an Au^I^ rather than an Au^III^ compound. The ratio of ppy to lpa is altered again, this time with a 1 : 1 ratio, resulting in the complex [Au(ppy)(lpa)] as depicted in Fig. 3. Unlike the other two complexes, no hydride signal was detected, and only one thiol proton was observed. Furthermore, the pyridyl-proton signals are downfield-shifted and overlap with the phenyl ring signals, indicating a geometry change from the square-planar Au^III^ to the linear Au^I^ complex. The formation of this Au^I^ intermediate clearly highlights the reduction and oxidation processes which lead to the formation of such complexes.

### Investigation of the reduction mechanism with lipoic acid

**Fig. 4:**
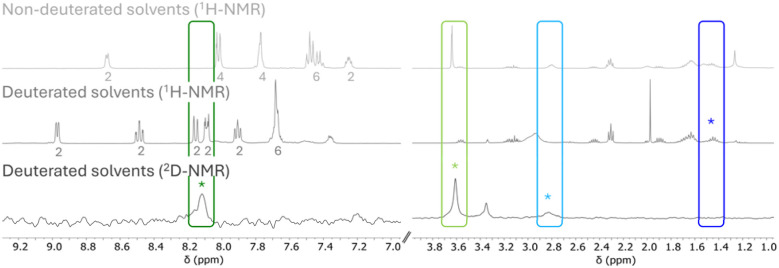
NMR spectra comparison of the reaction in non-deuterated (exemplarily for [Au_2_H_2_(ppy)_2_(lpa)_3_]) and deuterated solvents as well as the corresponding ^2^D NMR spectrum. The signals are color-coded corresponding to Fig. 3 with the asterisk marking the occurrence of the ppy-H, Au-H, thiol and methyl ester signals either the ^1^H or ^2^D NMR spectrum. For the aromatic region, the number of protons per signal is given.

To shed further light on the mechanism, additional experiments were conducted. First, the reaction was performed in deuterated solvents to establish the origin of the new aromatic proton, the methyl group, and the hydride from the thiols or solvent. For this purpose, the crude reaction mixture was directly measured to investigate changes compared to the previous NMR spectra. Additionally, a ^2^D NMR spectrum was recorded after switching to non-deuterated solvents. The spectral comparison is depicted Fig. 4 for [Au_2_H_2_(ppy)_2_(lpa)_3_].

Performing the reaction in deuterated solvents reduces the number of aromatic protons to 16, in turn the signal for the new ppy-D appears at 8.12 ppm in the ^2^D NMR spectrum. Additionally, the methyl group at 3.64 ppm disappears in the ^1^H NMR spectrum of the reaction with deuterated solvents and can instead be found in the ^2^D NMR. Interestingly, the Au-D signal is present in the ^2^D NMR at 2.83 ppm, while the thiol signals were found at 1.45 ppm in the ^1^H NMR. This indicates that the metal hydride bond is more stable than the S–H bond towards H/D exchange even in protic solvents. These results support the assumption of lpa esterification with methanol and confirm the participation of CD_3_OD or the thiol after H/D exchange in the Au–C bond cleavage and hydride formation.

To identify the initial reaction steps, the reaction time was decreased from 16 hours to 3 hours. During this time, the [Au(ppy)Cl_2_] must have reacted as it was completely dissolved. Interestingly, only lpa^ox^ was found but no gold lpa complex.

Combining all the results, the first part of the reaction mechanism is proposed in Scheme 1. The reaction starts with ligand exchange of a chloride to a thiolate of dihydro lipoic acid and the simultaneous liberation of hydrogen chloride (see Scheme 1(i)). By comparison with the cysteine (Cys) containing gold complex [Au(ppy)(Cys)](NO_3_) reported in the literature,^24^ this ligand exchange is assumed to occur in the *trans* position to the pyridyl nitrogen.

Expected from the work previously done in our group, the next step would be the deprotonation of the second thiol with subsequent metal coordination.^25^ However, in the absence of the peptide backbone, lpa oxidizes to the thermodynamically stable five-membered ring, which was confirmed as the disulfide form was the only detected lpa species after three hours. Therefore, the released hydride is then transferred to the Au^III^ complex, substituting the thiolate as the negatively charged ligand. Bochmann *et al.* showed that in binuclear bonded Au^III^ hydride complexes, the hydride binds *trans* to the carbonyl–gold bond.^22^ Consequently, we believe that in our reaction the *trans* position to the Au–C bond should also be favored (Scheme 1(ii)).

From complex 2, the C–H bond cannot be reductively eliminated as the hydride and carbanion are spatially separated. This strongly suggests that the Au–H is donated by lpa-H_2_. The formed hydride complex 2 is reactive, but the reverse reaction back to complex 1 is disfavored because the stable five-membered ring in oxidized lpa would have to be opened up again.

However, Au^I^ complexes have been reported to undergo oxidative addition with C–C bonds^26^ and even with the higher analogue Si–Si bonds,^27^ using conditions similar to those in this work. Even more strikingly, Gray and co-workers showed that the oxidative addition of a disulfide to Au^I^ and the reductive elimination of two thiols from Au^III^ are reversible processes for their studied example.^28^

To facilitate the reduction of the complex 2, a reductive elimination is necessary. However, the *trans* positioning of the carbanion and hydride impedes this reaction. Consequently, a ligand exchange must occur, replacing the chloride with a second hydride, which can be donated by lpa^red^ or methanol. lpa^red^ was employed in a 1 : 1 ratio with the [Au(ppy)Cl_2_] complex and is presumably consumed during the first reaction step, leaving only a small amount of hydride. On the other hand, methanol oxidation to formaldehyde could also occur. Although methanol is an extremely mild reductant, alcohols are well-established reductants for metal complexes and its interactions with Au^III^ could facilitate the reaction. In a cellular environment, however, the hydride donor is more likely lpa, NAD(P)H, or a similar biological reductant.

As a result of the hydride transfer, complex 3 then has a hydride in the *cis* position to the carbanion, enabling reductive elimination with C–H bond formation. Such C–H bond formations through oxidative addition and reductive elimination reactions are well-established processes with gold complexes as catalysts.^22,29^ This process is further favored because the hydride opposite the Au–C bond exerts a strong *trans*-effect, resulting in the formation of 5, which is a linear Au^I^ species. This complex is now able to undergo oxidative addition with the oxidized cyclic lipoic acid, forming a complex chelated by both thiolates of lpa (complex 6). It is most likely that esterification of the carboxylic acid (Scheme 1(iii)) occurred before addition to 5. The HCl produced by the ligand exchange reaction is highly reactive and, in the DCM/MeOH mixture, is strong enough to catalyze the esterification with methanol at room temperature. As a result, the methanol ester of lipoic acid 4 reacts with the Au^I^ complex 5.

**Fig. 5:**
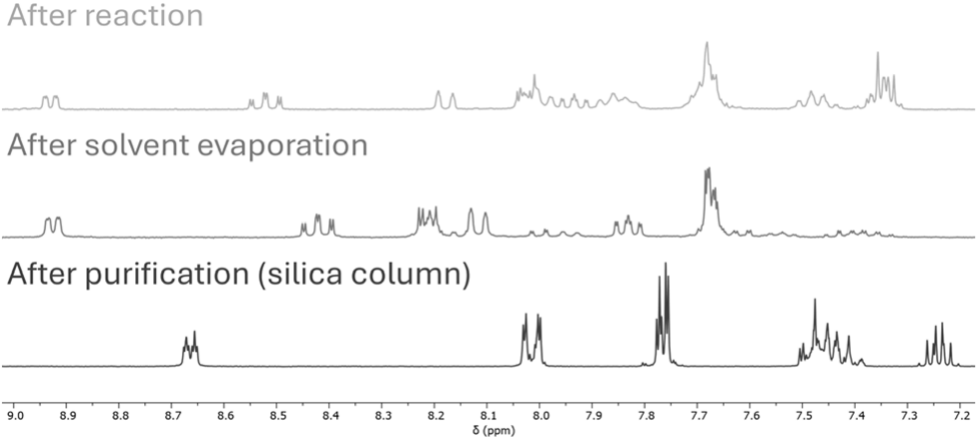
NMR spectra of the reaction in deuterated solvents directly after the 16 h reaction (top), after solvent evaporation (middle) and purification by filtration over a silica column (bottom).

With this mechanism, the occurrence of the unexpected additional protons of the ppy ligand, the Au–H, and the ester signal can be explained. However, the observed complexes are still not completely explained by this mechanism. To monitor further changes, NMR spectra were recorded at each step in the synthesis and purification process: before solvent evaporation, after solvent evaporation, and after silica column chromatography. As shown in Fig. 5, the formation of 6 in the crude reaction mixture is clearly proven. However, solvent evaporation at 40 °C alters the complex structure, and only after the purification the product NMR spectrum is obtained. Hence, complex 6 undergoes further changes during purification.

### Formation of polynuclear species

**Scheme 2:**
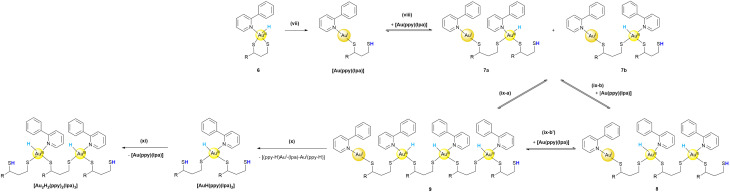
Proposed mechanism (part II) for the formation of the obtained products [Au(ppy)(lpa)], [Au_2_H_2_(ppy)_2_(lpa)_3_] and [AuH(ppy)(lpa)_2_] *via* reductive elimination and oxidative addition reactions, colour coding as in Fig. 1. (vii) Reductive elimination of the S–H bond initiated during purification, (viii) dimerization of [Au(ppy)(lpa)] by oxidative addition of S–H in a non-concerted (7a) or concerted (7b) fashion, (ix-a) direct oxidative addition of 7a and 7b or (ix-b) and (ix-b′) two times oxidative addition of [Au(ppy)(lpa)], (x) reductive elimination of [(ppy-H)Au^I^-(lpa)-Au^I^(ppy-H)] towards [Au_2_H_2_(ppy)_2_(lpa)_3_], followed by reductive elimination (xi) of [Au(ppy)(lpa)] yielding [AuH(ppy)(lpa)_2_].

For this reason, we propose a mechanism for the second part (see Scheme 2) towards the characterized products, which is initiated by either heat or interaction with the silica column material. The following reaction steps are a cascade of different reductive elimination and oxidative addition steps, which eventually explain the formation of all the observed intermediates shown in Fig. 3.

First, one thiol (RS-H) is reductively eliminated from 6, resulting in the isolated complex [Au(ppy)(lpa)] (Scheme 2(vii)). This aligns with the findings of Bochmann *et al.*, who reported that Au^I^ species originating from Au^III^ hydrides by C–H elimination can be isolated.^22^ Assuming that the reductive elimination of S–H is a reversible process, the oxidative addition of an S–H bond to a second molecule of [Au(ppy)(lpa)] is possible (step (viii)). Again, a reaction with 4 is not reasonable as an equimolar ratio of [Au(ppy)Cl_2_] and lpa^red^ was used and the lipoic acid derivatives are assumed to be fully converted during each reaction step. The addition can take place in a non-concerted (ix-a) or concerted (ix-b) fashion. Further oxidative additions of free thiols to other Au^I^-complexes lead to polymerization (see steps (ix)). Polymerization is a process often observed under acidic conditions for Au^I^ species, but usually led to Au–S–Au bridged complexes when monodentate ligands were used.^30^

In contrast to synthesis procedures for Au^III^-hydride complexes in the literature, the reactions in this work were performed at room temperature rather than at low temperatures. For the previously reported complexes, rearrangements and reductive eliminations are favored above 228 K.^22^ Consequently, ligand rearrangements are probable for polymeric structures like 9. Positional changes of the monodentate ligands, the hydride and ppy are likely. After the rearrangement, reductive elimination is not only possible as a reverse reaction, but also a new complex can be formed. Step (x) shows the chain breakdown that decreases the length of the polymeric structure, which is entropically favored as more and smaller molecules are obtained. This results in the experimentally obtained [Au_2_H_2_(ppy)_2_(lpa)_3_] complex, which can subsequently undergo a final reductive elimination of an S–H bond yielding [AuH(ppy)(lpa)_2_], which was isolated and characterized.

The proposed mechanism explains all ^1^H-NMR signals for all detected complexes. Moreover, it aligns with the experimental observation that [Au(ppy)(lpa)] was only observed once, [Au_2_H_2_(ppy)_2_(lpa)_3_] several times and [AuH(ppy)(lpa)_2_] was obtained predominantly as the most stable product. Nonetheless, all compounds are still very unstable and cannot be stored in solution or dried. Consequently, additional characterization methods, such as elemental analysis and X-ray single crystal structures, could not be employed.

### Reaction with cysteine derivatives

To determine whether lipoic acid possesses unique properties in the reduction of Au^III^ through the formation of the five-membered ring, *N*-acetyl-l-cysteine-methyl ester (NAC-OMe) was employed as a second naturally relevant reductant. The protection of both sites was selected to mimic the properties of glutathione (GSH) and to minimize amine coordination.

The reaction was carried out similarly to the lipoic acid experiments, but with either one or two equivalents of NAC-OMe. One equivalent corresponds to the same amount of ligand, while two equivalents equate to the number of reduction equivalents in lpa. The reaction mixtures were analysed directly without purification.

**Fig. 6:**
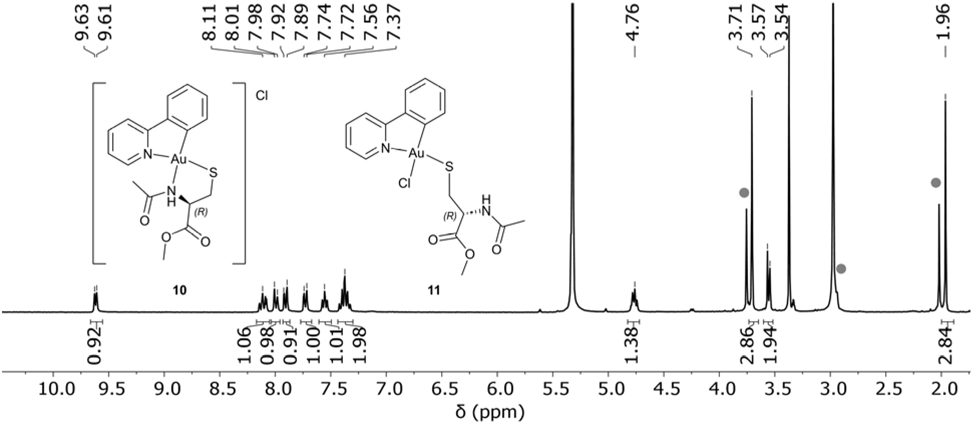
^1^H NMR spectrum of the [Au(ppy)Cl_2_] NAC-OMe reaction mixture in CD_2_Cl_2_/CD_3_OD with the two possible binding modes for *N*-acetyl-cysteine-methyl ester acting as a N^S-bidentate ligand (10) or as a monodentate thiolate ligand (11). The signals marked with grey circles correspond to the free ligand.

Unexpectedly, unreacted NAC-OMe was found in both reaction mixtures. This contrasts with the crude NMR of the lipoic acid reaction and suggests a different reaction mechanism. The formed complex and the ligand were distinguished and assigned using a DOSY spectrum. The ^1^H NMR spectrum (see Fig. 6) reveals an intact Au–C bond, indicated by the presence of eight aromatic proton signals. NAC-OMe and ppy were found in the same ratio, which was for the lpa complexes an exceptional case for the Au^I^ complex 5.

The NMR analysis allows two possible binding modes, depicted in Fig. 6. The complex could be similar to 2, with cysteine acting as a monodentate ligand binding through the thiolate (11). Alternatively, it could bind in a bidentate N^S fashion (10). This would align with the structures published by Konno *et al.*^24^ and Lee *et al.*,^31^ where they synthesised mononuclear Au^III^ complexes in aqueous solutions with cysteine derivatives acting as chelating ligands. The signal in ESI-MS at *m* = 585.4 *m*/*z* matches the mass of [11 + Na]^+^.

**Fig. 7:**
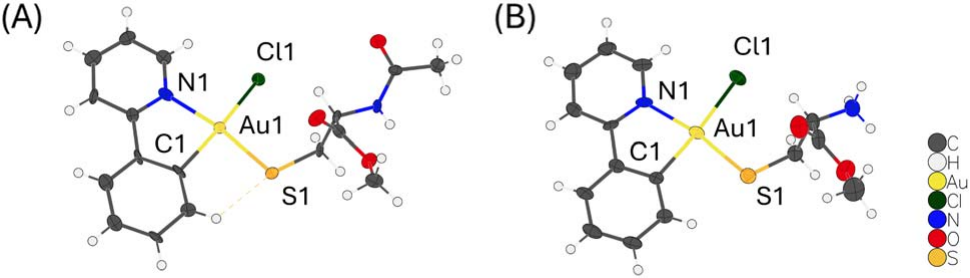
ORTEP-plot of 11 (A) and [AuCl(ppy)(HC–OMe)] (B). Ellipsoids are drawn at the 50% probability level. For [AuCl(ppy)(HC–OMe)], the solvent and counter ion are omitted for clarity.

Moreover, single crystals of the square-planar gold complex 11 were obtained after several days, indicating greater stability compared to the lpa complexes. The crystal structure also supports the monodentate binding mode (Fig. 7A). Originating from the bidentate ppy ligand, which forms a five-membered ring with Au, the square planar structure is slightly distorted. The C–Au–N angle is only 81.38°, while the S–Au–Cl angle is extended to 96.40°. The *trans*-effect is evident, as the Au–C bond is shortened to 2.062 Å, whereas the Au–Cl bond is elongated to 2.377 Å. The position of the thiolate *trans* to the pyridyl-N aligns with the proposed first step in the binding cascade of lipoic acid, further supporting the mechanism (step (i) in Scheme 1). Additionally, these results show that C–S cross coupling using Au^III^ complexes requires bridged C^N ligands,^17^ and is not a general mode of action.

Additionally, single crystals with cysteine-methyl ester (HC-OMe) were grown to investigate whether the binding difference arises from the synthesis conditions or the less electron-rich acetyl-protected nitrogen. The crystal structure displays the same monodentate binding mode as with NAC-OMe, with matching square-planar geometry, bond lengths, and angles (Fig. 7B). In contrast to its acetyl-protected analogue, the free amine is protonated due to the presence of HCl released during the reaction. The resulting positive charge prevents bidentate coordination, which was observed in aqueous media, and instead promotes monodentate binding. This highlights the different possibilities of cysteine–gold binding, which are strongly influenced by the solvent environment.

Remarkably, all cysteine derivatives bind to the gold metal but only lpa is able to reduce Au^III^ to Au^I^. The redox properties of lpa and the essential antioxidant GSH were measured elsewhere at 25 °C and pH 7, yielding standard reduction potentials of *E*_0_ = −0.29 V and *E*_0_ = −0.23 V, respectively.^32^ These values demonstrate that lpa is the stronger reductant^33^ and is capable of reducing Au^III^, whereas GSH or its derivatives primarily act as ligands, binding to the gold complex without reducing it.

It should be noted that this work was performed under degassed conditions/N_2_ protection to ensure a high concentration of reduced lpa at all times. However, the presence of oxygen in biological systems is not assumed to change our conclusions as lpa can then be constantly reduced by the cellular machinery.

## Experimental

### Materials and methods

All chemicals and solvents were purchased from commercial suppliers and used without further purification. Solvents were degassed with nitrogen for 10 min prior to use or used as received. ^1^H NMR and ^13^C{^1^H} NMR measurements were conducted at 298 K on a Bruker AV-III 300, and residual solvent signals from the deuterated solvents were used to reference the ^1^H or ^13^C signals. MestReNova was used to perform spectrum analysis, signals were reported in parts per million (ppm) with coupling constants (*J*) given in Hz. The numbering scheme for signal assignment is found in the SI. Electron spray ionization mass spectra were recorded using a Bruker Esquire 6000 in the positive sensitivity mode. For single-crystal diffraction studies, a Synergy S dual S3 wavelength diffractometer employing mirror monochromatic CuKα radiation generated from a PhotonJet-S (Cu) X-ray Source with a HyPix-600 HE detector was used for data collection. Cell constants were obtained from a least square's refinement. CrysAlisPro was used for integration, reduction, and absorption correction of the data, measured at 100.15 K. The structures were solved using SHELXT 2018/2 (ref. 34) and refined using SHELXL 2018/3.^35^ The Olex2 1.3 (ref. 36) interface was used to generate publication material.

### Synthetic procedures

[Au(ppy)Cl_2_] was synthesised according to the literature procedure.^37^ Tetrachloroauric acid (0.05 g, 0.13 mmol, 1.0 eq.) was dissolved in 4 mL water, and 2-phenylpyridine (0.02 mL, 0.14 mmol, 1.1 eq.) was added. Immediately, some solid precipitated. The solution was irradiated for one hour in a microwave (55 W, 170 °C, 13 bar). The formed solid was filtered off, washed with water (3 × 2 mL) and dried for 1 h under a N_2_ stream. The product was obtained as a light-yellow solid. Yield: 0.03 g (0.08 mmol, 63%). ^1^H NMR (300 MHz, DMSO-*d*_6_): *δ* (ppm) 9.53 (1H, d, H_A_, *J*_HA−HB_ = 6.10 Hz), 8.42 (2H, m, 1xH_C_, 1xH_D_), 7.98 (1H, d, H_E_, *J*_HE−HF_ = 7.52 Hz), 7.82 (1H, d, H_H_), 7.77 (1H, td, H_B_, *J*_HB−HA_ = 6.20 Hz), 7.48 (1H, t, H_F_, *J*_HF−HE_ = 7.47 Hz), 7.38 (1H, t, H_G_).

#### lpa^red^ (dihydrolipoic acid)

Lipoic acid (0.10 g, 0.48 mmol, 1.0 eq.) was dissolved in 6 mL MeCN/H_2_O (3 : 1) and tris(2-carboxy ethyl)phosphine hydrochloride (TCEP) (0.28 g, 0.97 mmol, 2.0 eq.) was dissolved in 3 mL MeCN/H_2_O (3 : 1). Both solutions were degassed with N_2_ for 10 min. TCEP was added to the yellow lpa solution, which was decolorized within 5 min. The mixture was stirred for one hour at room temperature, and the aqueous phase was extracted with DCM (3 × 10 mL). After drying the combined organic phases over MgSO_4_, the solvent was removed under reduced pressure, resulting in a colorless liquid. Yield: 0.08 g (0.37 mmol, 77%). ^1^H NMR (300 MHz, CDCl_3_): *δ* (ppm) 10.98 (1H, s, COOH), 2.96–2.85 (1H, m, H_g_), 2.78–2.58 (2H, m, H_i_), 2.36 (2H, t, H_c_), 1.94–1.83 (1H, m, H_h_), 1.78–1.71 (1H, m, H_h_), 1.69–1.39 (6H, m, 2xH_d_, 2xH_e_, 2xH_f_), 1.34 (1H, t, SH_i_), 1.30–1.28 (1H, d, SH_g_). ^13^C{^1^H} NMR (75 MHz, CDCl_3_): *δ* (ppm) 179.6 (C=O), 42.9 (C_c_), 39.4 (C_SH_), 38.9 (C_SH_), 34.0 (C_h_), 26.6 (C_aliph_), 24.4 (C_aliph_), 22.4 (C_aliph_).

#### General procedure for [Au_*x*_(ppy)_*x*_(lpa)_*y*_] complexes

[Au(ppy)Cl_2_] (0.02 g, 0.05 mmol, 1.0 eq.) was suspended in 30 mL DCM/MeOH (9 : 1) and degassed with N_2_ for 10 min, to maintain the reduced form of lpa in the subsequent reaction. A solution of dihydro lipoic acid (0.01 g, 0.05 mmol, 1.0 eq.) in degassed DCM/MeOH (9 : 1) was added slowly, resulting in an immediate change from colorless to yellow. After stirring the solution under N_2_ for 16 h at room temperature, the solvent was removed under reduced pressure. The solid was purified by column chromatography on silica (10 mL), eluting with *n*-hexane/EtOAc. The product was obtained as a yellow-brownish oil after removing the solvent under reduced pressure.

#### [Au(ppy)(lpa)]

Yield: 7.10 mg (12.38 µmol, 25%). *R*_f_ = 0.62 in *n*-hexane/EtOAc (2 : 1). ^1^H NMR (300 MHz, CDCl_3_): *δ* (ppm) 8.83 (1H, d, H_A_), 8.08 (2H, m, 1xH_E_, 1xH_M_), 8.02 (1H, d, H_C_), 7.89 (1H, d, H_D_), 7.59–7.46 (4H, m, 1xH_F_, 1xH_G_, 1xH_H_, 1xH_B_), 3.67 (3H, s, COOMe) 3.57 (1H, q, H_g_), 3.23–3.07 (2H, m, H_i_), 2.46 (1H, sex, H_h_), 2.33 (2H, t, H_c_), 1.91 (1H, sex, H_h_), 1.74–1.61 (4H, m, 2xH_d_, 2xH_f_), 1.52–1.40 (3H, m, 1xSH, 2xH_e_).

#### [Au_2_H_2_(ppy)_2_(lpa)_3_]

Yield: 4.80 mg (3.51 µmol, 22%). *R*_f_ = 0.54 in *n*-hexane/EtOAc (4 : 1). ^1^H NMR (300 MHz, CDCl_3_): *δ* (ppm) 8.79 (2H, d, H_A_), 8.06 (4H, d, 2xH_E_, 2xH_M_), 7.95 (2H, t, H_C_), 7.84 (2H, d, H_D_), 7.56–7.48 (6H, m, 2xH_F_, 2xH_G_, 2xH_H_), 7.40 (2H, t, H_B_), 3.67 (9H, s, COOMe), 3.57 (3H, q, H_g_), 3.23–3.07 (6H, m, H_i_), 2.78 (2H, s, Au–H), 2.46 (3H, sex, H_h_), 2.33 (6H, t, H_c_), 1.90 (3H, sex, H_h_), 1.74–1.61 (12H, m, 6xH_d_, 6xH_e_), 1.52–1.42 (8H, m, 6xH_f_, 2x SH). ESI-MS (pos): m/2z 685.2, calc. for [C_49_H_70_Au_2_N_2_O_6_S_6_]^2+^ = 685.2.

#### [AuH(ppy)(lpa)_2_]

Yield: 4.85 mg (6.10 µmol, 25%). *R*_f_ = 0.58 in *n*-hexane/EtOAc (4 : 1). ^1^H NMR (300 MHz, CDCl_3_): *δ* (ppm) 8.76 (1H, d, H_A_), 8.03 (2H, d, 1xH_E_, 1xH_M_), 7.89 (1H, t, H_C_), 7.81 (1H, d, H_D_), 7.53–7.44 (3H, m, 1xH_F_, 1xH_G_, 1xH_H_), 7.35 (2H, t, H_B_), 3.67 (6H, s, COOMe), 3.57 (2H, quin, H_g_), 3.22–3.06 (4H, m, H_i_), 2.78 (1H, s, Au–H), 2.45 (2H, sex, H_h_), 2.32 (4H, t, H_c_), 1.92 (2H, sex, H_h_), 1.73–1.61 (8H, m, 4xH_d_, 4xH_f_), 1.57–1.36 (6H, m, 2xSH, 4xH_e_). ^13^C{^1^H} NMR (75 MHz, CDCl_3_): *δ* (ppm) 174.1 (C=O), 129.3 (C_arom_), 127.5 (C_arom_), 122.9 (C_arom_), 56.5 (C_c_), 51.7 (C_b_), 40.4 (C_SH_), 38.6 (C_SH_), 34.7 (C_aliph_), 34.0 (C_aliph_), 28.9 (C_aliph_), 24.8 (C_aliph_).

#### General procedure for [Au(ppy)(XC-OMe)] complexes

[Au(ppy)Cl_2_] (15 mg, 0.04 mmol, 1.0 eq.) was suspended in 10 mL DCM/MeOH (9 : 1) and degassed with N_2_ for 10 min. A solution of cysteine derivative (12.6 mg, 0.07 mmol, 2.0 eq. or 6.3 mg, 0.04 mmol, 1.0 eq.) in 0.5 mL degassed DCM/MeOH (9 : 1) was added slowly, resulting in a color change from colorless to yellow within 5 minutes. After stirring the solution under N_2_ for 17 h at room temperature, the solvent was removed under reduced pressure. NMR spectra of the crude yellow products were measured without purification.

#### [AuCl(ppy)(NAC-OMe)] (**11**)

^1^H NMR (300 MHz, CD_2_Cl_2_): *δ* (ppm) 9.61 (1H, d, H_A_), 8.11 (2H, m, H_arom_), 7.99 (1H, d, H_arom_), 7.90 (1H, dd, H_arom_), 7.58–7.53 (1H, m, H_arom_), 7.42–7.32 (2H, m, H_arom_), 4.77 (1H, q, H_b′_), 3.70 (3H, s, H_d′_), 3.55 (2H, dd, H_c′_), 1.96 (3H, s, H_a′_). ESI-MS (pos): *m*/*z* 485.4, calc. for [C_15_H_16_AuN_2_O_2_S]^+^ = 485.1; *m*/*z* 527.4, calc. for [C_17_H_18_AuN_2_O_3_S]^+^ = 527.1; *m*/*z* 585.4, calc. for [C_17_H_18_AuClN_2_NaO_3_S]^+^ = 585.0. XRD: orthorhombic, *P*2_1_2_1_2_1_, 4.8602(1), 14.2036(2), 25.7174(4), 90, 90, 90, 1775.33(5).

#### [AuCl(ppy)(HC-OMe)]

XRD: monoclinic, *P*2_1_, 17.2761(3), 6.74540(10), 18.9344(3), 90, 115.370(2), 90, 1993.71(6).

## Conclusions

Beyond the almost ubiquitous Pt anticancer compounds, a few Au complexes in the oxidation state +I are in clinical use. In addition, also Au^III^ complexes were suggested as promising drug candidates. It is generally assumed that intracellular reduction activates the Au^III^ complexes to their biologically active Au^I^ analogues, however details remain elusive. Herein, we demonstrate a pathway for the reduction of Au^III^ to Au^I^ complexes using a biological relevant reductant, by isolation and characterization of several intermediates by NMR spectroscopy and MS. The reaction mechanism involves Au^III^ hydrides, and a series of oxidative addition and reductive elimination reactions occurs. Noteworthy, Au hydrides (which were previously generated only under rigorously dry conditions at temperatures below 0 °C)^22^ were observed in this work in a protic solvent at room temperature.

Essential for the gold reduction is the use of naturally abundant lipoic acid (lpa) instead of GSH derivatives on our tested system. It is noteworthy that the reaction with simple thiols, such as cysteine methyl ester and its *N*-acetylated analogue (NAC-OMe), does not yield the same reduction products under identical conditions. While in all cases, thiol coordination on the Au^III^ center is the first step, only lpa will induce further reduction of the Au center through formation of Au hydrides. This suggests that the key factor is the slightly more negative reduction potential of lpa,^32,33^ and possibly also the entropically favoured formation of cyclic products in the case of lpa only. Therefore, we recommend that lpa should be considered in stability tests involving redox-sensitive complexes, to ensure that the observed activity is correctly attributed to the appropriate complex and metal oxidation state. Additionally in this work, the formation of di- and polymeric Au-clusters suggests a route for intracellular Au^III^ reduction, which could be the first steps towards the formation of gold nano particles – a side reaction that is often observed in gold chemistry.

Finally, our work bears important implications for the future design of Au^III^ complexes in medicinal inorganic chemistry. Previous work has already suggested that Au^III^ compounds are prodrugs which are converted into the active species by an activation-by-reduction mechanism. With a more detailed, mechanistic view from this work on the exact reduction mechanism and Au hydride species as possible intermediates, it is now possible to design Au^III^ prodrugs in a more rational way. Also, the importance of matching redox potentials in Au complexes to biological reductants becomes apparent. And lastly, a paradigm arises for the design of redox-inert Au^III^ complexes. In such compounds, hydride formation and particularly reductive elimination from any possible Au–H intermediates should be prevented by ligand design.

## Author contributions

J. O.: investigation, formal analysis, writing – original draft; N. M.-N.: conceptualization, funding acquisition, supervision, writing – review & editing.

## Conflicts of interest

There are no conflicts to declare.

## Supplementary Material

SC-OLF-D5SC06212H-s001

SC-OLF-D5SC06212H-s002

## Data Availability

CCDC 2424404(11) and 2424406(12) contain the supplementary crystallographic data for this paper.^38*a*,*b*^ All other supporting data to this article is included in the Supplementary information (SI). Supplementary information is available. See DOI: https://doi.org/10.1039/d5sc06212h.
