# Supplementary material for: Identification of Au-hydrides as key intermediates in the reduction of Au(iii) prodrugs to active Au(i) species under protic conditions
Source: Chem Sci. 2026 Jan 7;17(8):4183–90. doi: 10.1039/d5sc06212h (PMC12777740; doi:10.1039/d5sc06212h)
Supplement: SC-017-D5SC06212H-s001 [file SC-017-D5SC06212H-s001.pdf]

Supplementary Information (SI) for Chemical Science.
This journal is © The Royal Society of Chemistry 2025

Supporting Information

SC-EDG-08-2025-006212

Identification of Au-hydrides as Key Intermediates in the Reduction of Au(III) Prodrugs to Active Au(I) Species under Protic Conditions

Jasmine Ochs, Nils Metzler-Nolte

Faculty of Chemistry and Biochemistry, Inorganic Chemistry I – Bioinorganic Chemistry, Ruhr University Bochum, Universitaetsstrasse 150, 44801 Bochum, Germany

E-mail: Nils.Metzler-Nolte@rub.de

Inhalt

1. NMR Spectra 3
   1.1. Numbering Schemes 3
   1.2. ^{1}H NMR Spectra and COSY 3
   1.3. ^{13}C{^{1}H} NMR Spectra 6
   1.4. DOSY 7
2. ESI-MS 8
3. Crystallographic Data 9
   3.1. Selected Bond Lengths and Angles 11

1. NMR Spectra

1.1. Numbering Schemes

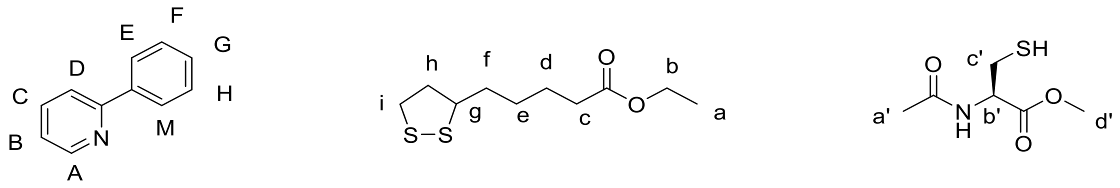

Figure A1: Numbering scheme used for the assignment of NMR-signals.

1.2. ^{1}H NMR Spectra and COSY

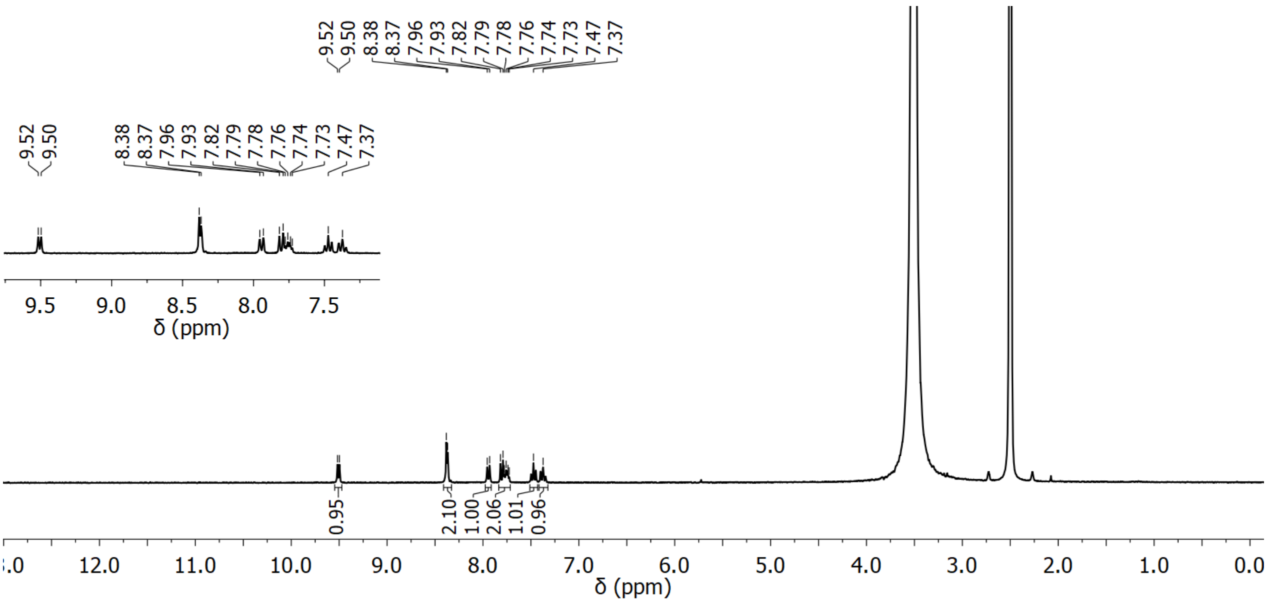

Figure A2: ^{1}H NMR spectrum of [Au(ppy)Cl$_2$] in DMSO-d$_6$ recorded at 300 MHz.

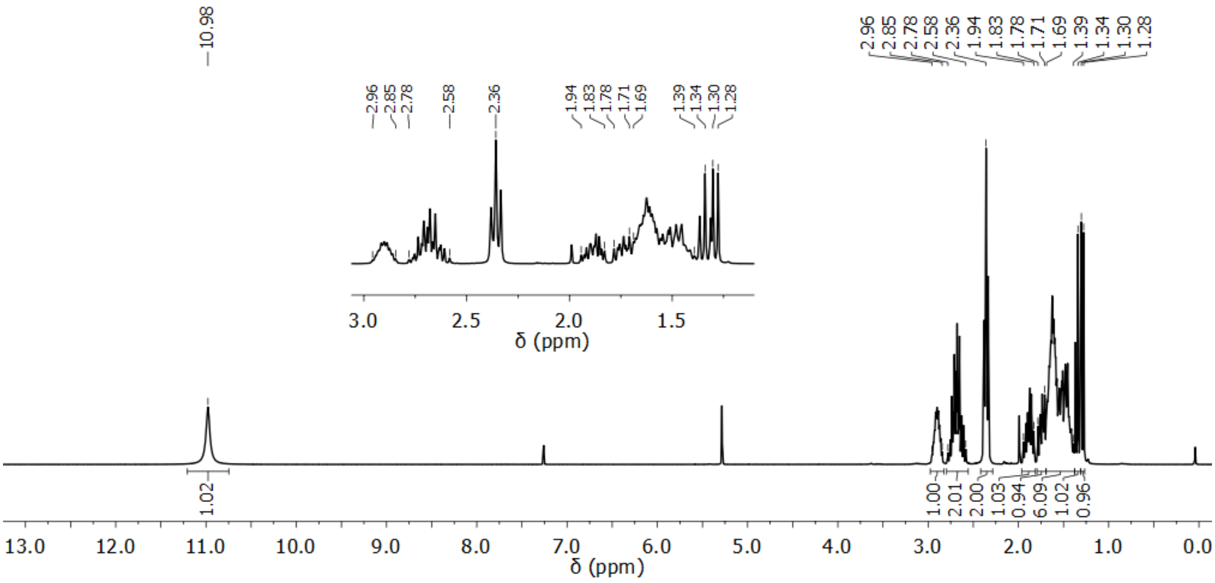

Figure A3: ^{1}H NMR spectrum of the lpa^{red} in $CDCl_3$ recorded at 300 MHz.

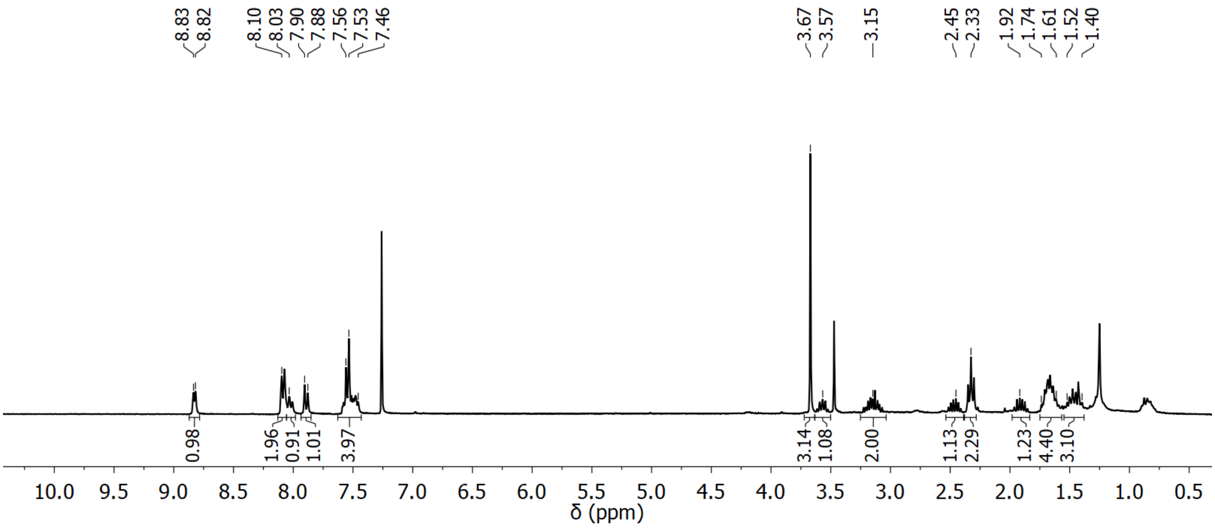

Figure A4: ^{1}H NMR spectrum of [Au(ppy)(lpa)] in $CDCl_3$ recorded at 300 MHz.

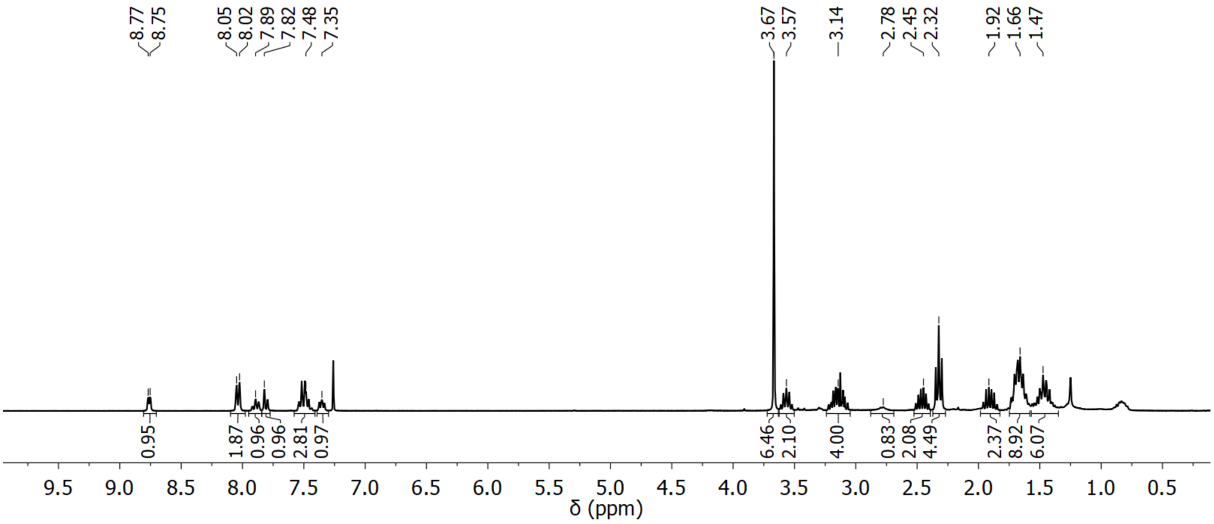

Figure A5: ^{1}H NMR spectrum of [AuH(ppy)(lpa)$_2$] in $CDCl_3$ recorded at 300 MHz.

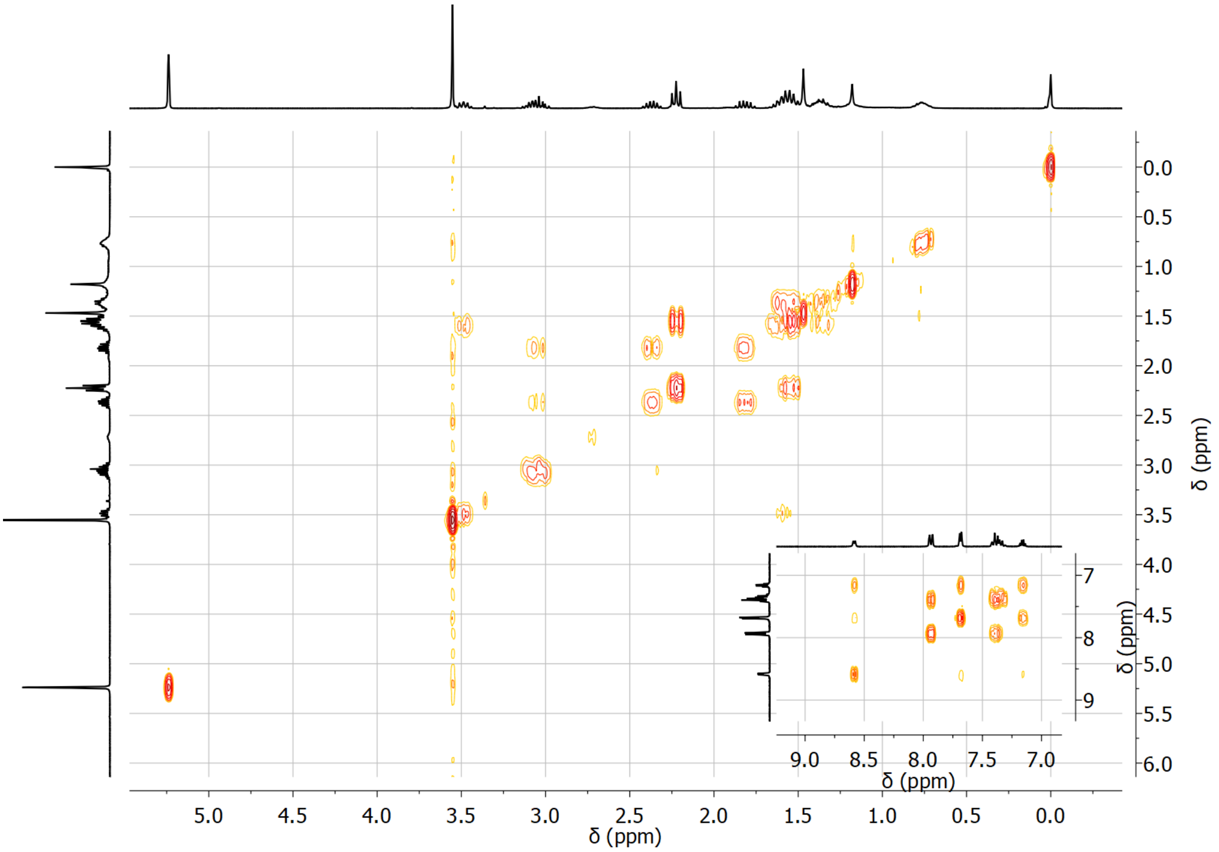

Figure A6: COSY spectrum of [AuH(ppy)(lpa)$_2$] in $CDCl_3$ recorded at 300 MHz.

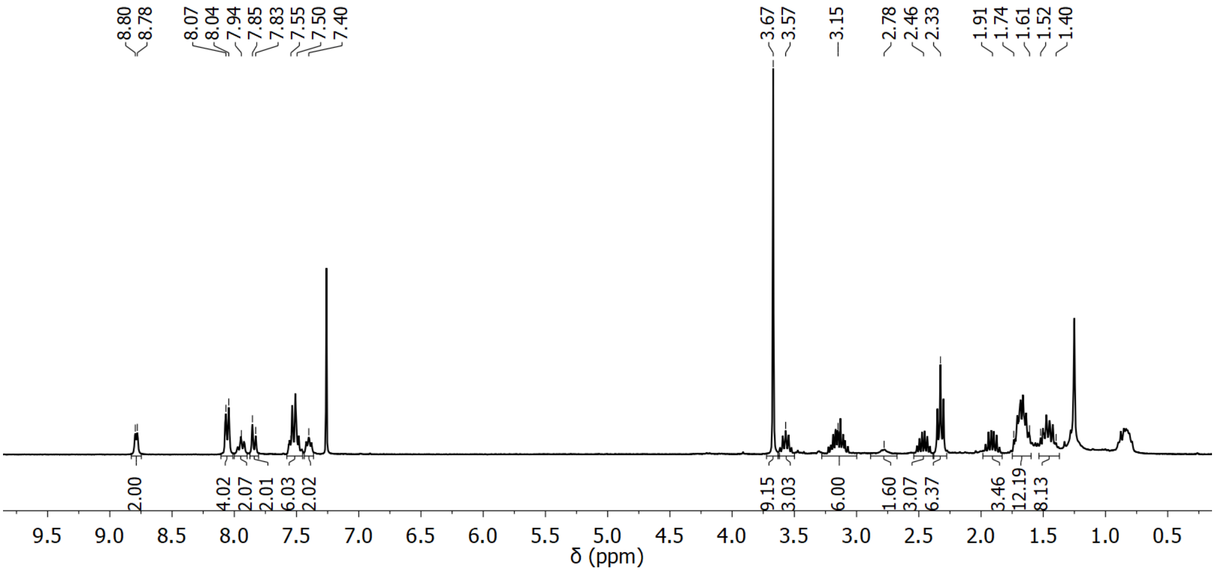

Figure A7: ^{1}H NMR spectrum of $[Au_2H_2(ppy)_2(lpa)_3]$ in $CDCl_3$ recorded at 300 MHz.

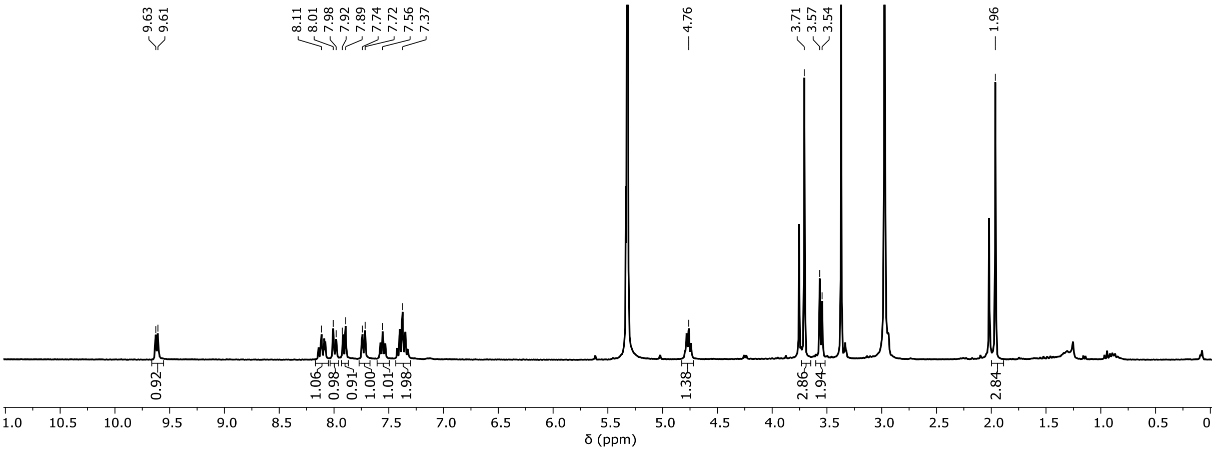

*Figure A 1: ^{1}H NMR spectrum of **11** in CD_2Cl_2/CD_3OD recorded at 300 MHz.*

1.3. $^{13}C\{^{1}H\}$ NMR Spectra

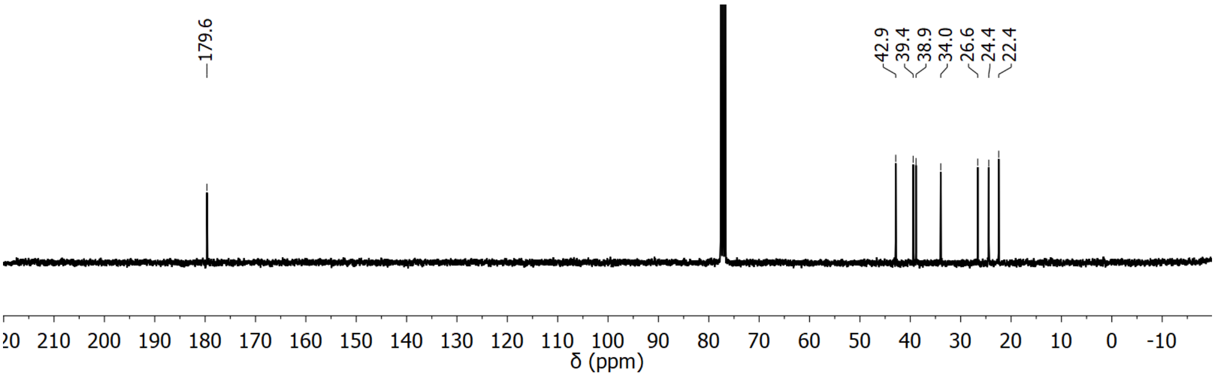

Figure A8: $^{13}C\{^{1}H\}$ NMR spectrum of the lpa^{red} in $CDCl_3$ recorded at 75 MHz.

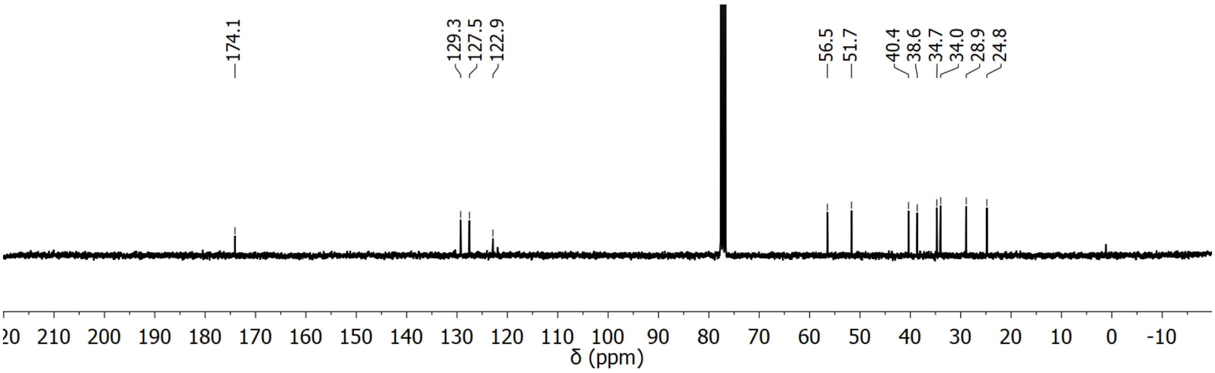

Figure A 9: $^{13}C\{^1H\}$ NMR spectrum of $[AuH(ppy)(lpa)_2]$ in $CDCl_3$ recorded at 75 MHz.

1.4. DOSY

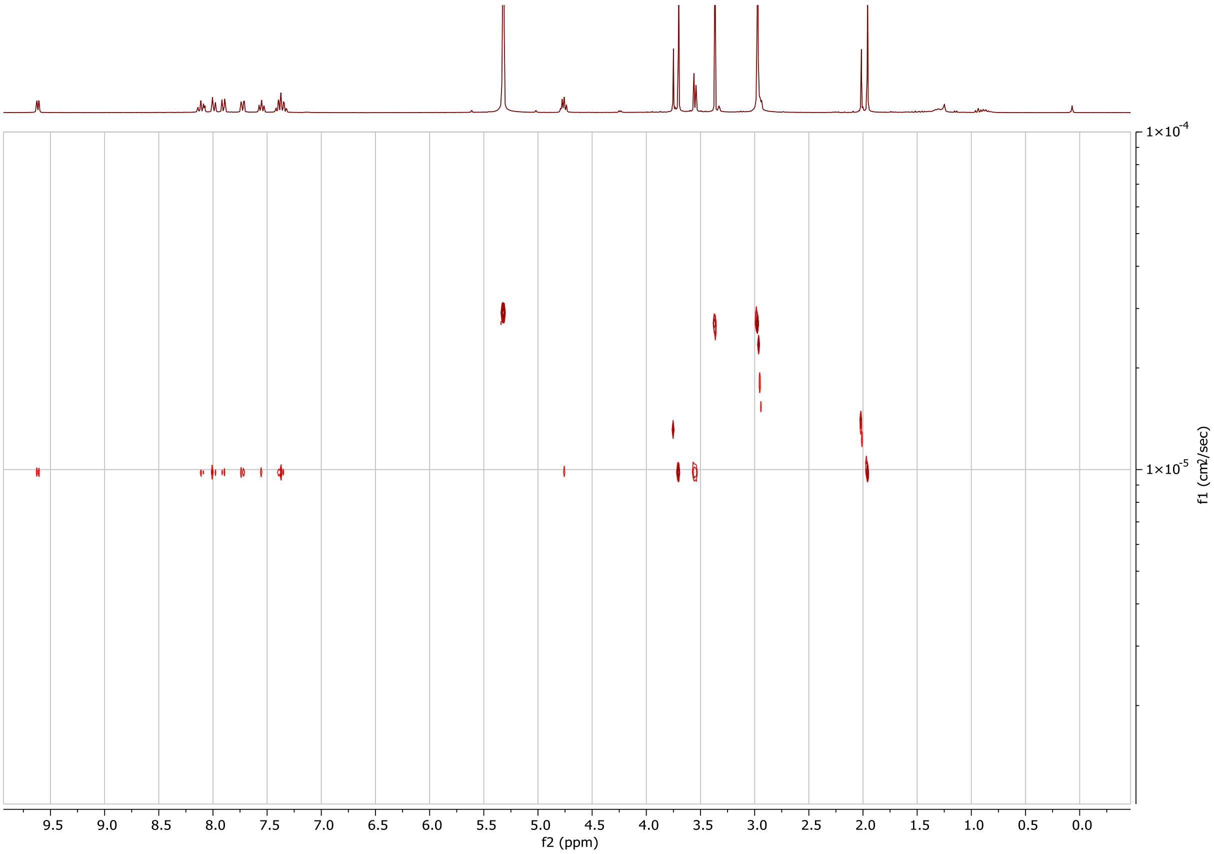

*Figure A 2: DOSY of **11** in CD_2Cl_2/CD_3OD recorded at 300 MHz to confirm peaks of the gold complex.*

2. ESI-MS

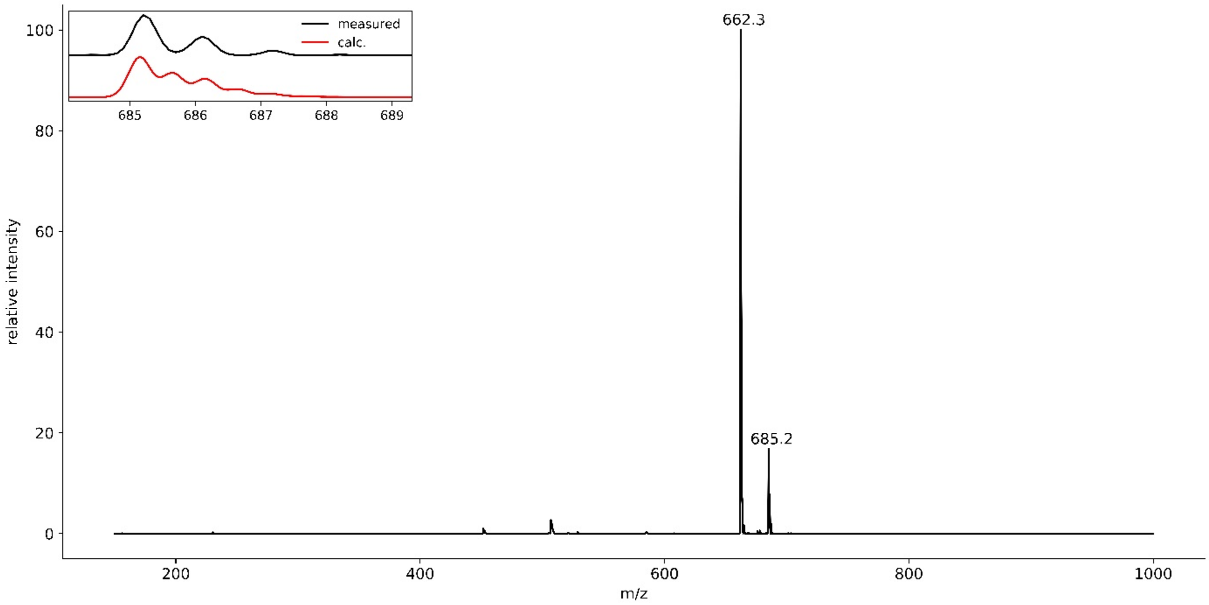

Figure A10: ESI-MS (pos.) of [Au_2H_2(ppy)$_2$(lpa)$_3$].

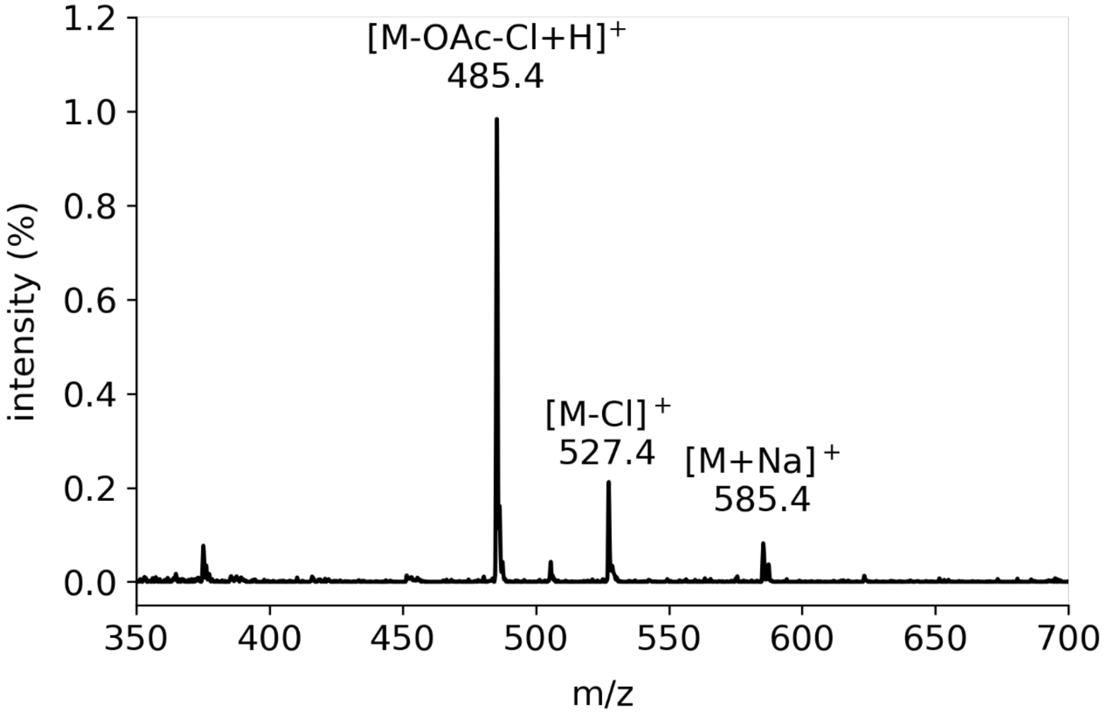

*Figure A 3: ESI-MS (pos.) of **11**.*

3. Crystallographic Data

*Table 1: Crystallographic Data of **11** crystallized by slow evaporation of DCM/methanol.*

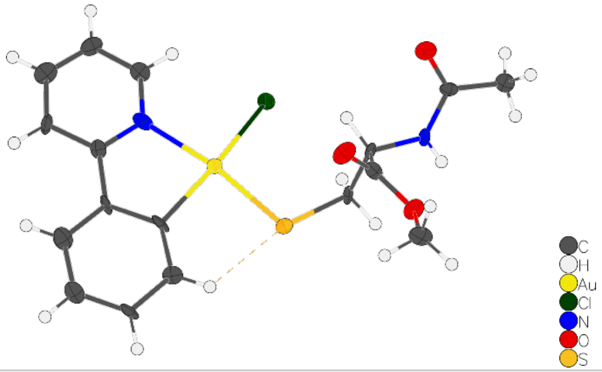

Empirical formula	$C_{17}H_{18}AuClN_2O_3S$
Formula weight [g/mol]	562.829
Temperature [K]	106(1)
Wavelength [Å]	1.54
Crystal system	Orthorhombic
Space group	$P2_12_12_1$
Unit cell	a = 4.8602(1) α = 90<br>b = 14.2036(2) β = 90<br>c = 25.7174(4) γ = 90
Volume [Å^3]	1775.33(5)
Z	4
Density (calculated) [Mg/m^3]	2.106
F (000)	1070.6
θ range [°]	6.88 to 153.32
Reflections collected / unique	6668
Data / restraints / parameters	3154 / 12 / 226
Goodness-of-fit on F^2	1.020
R1/wR2 [I>2σ(I)]	R_1 = 0.0391, wR_2 = 0.1040
R1/wR2 (all data)	R_1 = 0.0409, wR_2 = 0.1055

Table 2: Crystallographic Data of [AuCl(ppy)(HC-OMe)] crystallized by slow evaporation of diethyl ether into DCM/methanol.

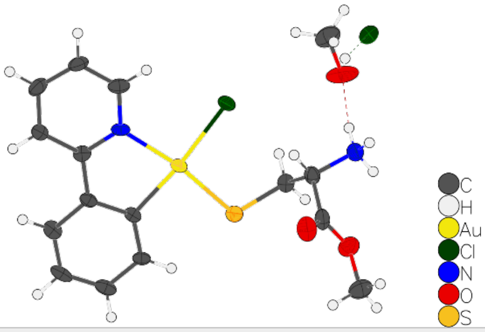

Empirical formula	$C_{32}H_{42}Au_2Cl_4N_4O_6S_2$
Formula weight [g/mol]	1178.589
Temperature [K]	100.0(3)
Wavelength [Å]	1.54
Crystal system	Monoclinic
Space group	$P2_1$
Unit cell	a = 17.2761(3) α = 90<br>b = 6.7454(1) β = 115.370(2)<br>c = 18.9344(3) γ = 90
Volume [Å^3]	1993.71(6)
Z	2
Density (calculated) [Mg/m^3]	1.963
F (000)	1128.1
θ range [°]	5.16 to 153.38
Reflections collected / unique	13529
Data / restraints / parameters	6100 / 1 / 472
Goodness-of-fit on F^2	1.039
R1/wR2 [I>2σ(I)]	R_1 = 0.0352, wR_2 = 0.0962
R1/wR2 (all data)	R_1 = 0.0365, wR_2 = 0.0969

3.1. Selected Bond Lengths and Angles

*Table 3: Selected bond lengths (Å) and bond angles (°) for **11** and **12**.*

	Au(ppy)(NAC-OMe) **11**	Au(ppy)(HC-OMe) **12**
Au1-C1	2.062(8)	2.039(6)
Au1-N1	2.103(8)	2.084(6)
Au1-S1	2.288(2)	2.2931(19)
Au1-Cl1	2.377(2)	2.3647(17)
Cl1-Au1-S1	96.40(7)	95.98(6)
S1-Au1-C1	89.0(3)	90.0(2)
C1-Au1-N1	81.4(3)	80.8(3)
N1-Au1-Cl1	93.2(2)	93.24(18)
Cl1-Au1-C1	170.8(3)	173.8(2)
S1-Au1-N1	170.3(2)	170.65(17)
